# Implicit attitudes towards older people among paid and unpaid caregivers of older people: a cross-sectional study

**DOI:** 10.1590/1980-5764-DN-2025-0337

**Published:** 2026-02-16

**Authors:** Madson Alan Maximiano-Barreto, Bruna Moretti Luchesi, Marcos Hortes Nisihara Chagas

**Affiliations:** 1 Universidade de São Paulo, Faculdade de Medicina de Ribeirão Preto, Departamento de Neurociências e Ciências do Comportamento, Grupo de Pesquisa em Saúde Mental, Cognição e Envelhecimento, Ribeirão Preto SP, Brazil.; 2 Universidade Federal do Mato Grosso do Sul, Três Lagoas MS, Brazil.

**Keywords:** Brazil, Caregivers, Cross-Sectional Studies, Attitude, Brasil, Cuidadores, Estudos Transversais, Atitude

## Abstract

Ageism is a type of prejudice that can be manifested implicitly or explicitly. Implicit ageism has been assessed in different populations (e.g., students and healthcare providers). This form of prejudice can exert an impact on social relationships and the offer of care, as many caregivers (e.g., those of older people) may work with individuals that are not of their interest for various reasons, which can have an impact on both mental health and the quality of care offered to others (e.g., older people).

**Objective:**

To assess implicit attitudes towards older people among paid and unpaid caregivers of older people.

**Methods:**

A cross-sectional study was conducted with 111 paid and unpaid caregivers of older people who reside in the city of São Carlos-SP, Brazil and surrounding region. The caregivers completed data collection instruments addressing implicit attitudes, the impact of providing care, depressive symptoms, sociodemographic characteristics and aspects of the care provided. Based on the score of the implicit attitude test, the caregivers were divided into three groups: “neutral implicit attitudes”, “positive implicit attitudes” and “negative implicit attitudes”.

**Results:**

Women accounted for the vast majority of the sample (91.0%) and age ranged from 19 to 76 years. A total of 63.1% of the paid and unpaid caregivers had negative implicit attitudes towards older people and only 12.6% had positive implicit attitudes.

**Conclusion:**

Caregivers of older people have negative implicit attitudes towards older people. Further studies are needed to investigate the impact of implicit attitudes on the caregiver-recipient relationship.

## Abstract (pt)

**RESUMO.:**

O etarismo é um tipo de preconceito que pode se manifestar de forma implícita ou explícita. O preconceito implícito vem sendo avaliado em diferentes populações (e.g., estudantes e profissionais da saúde). Esse preconceito pode impactar as relações sociais, assim como a prestação do cuidado, uma vez que muitos prestadores de cuidados (e.g., cuidadoras de pessoas idosas) podem trabalhar com público-alvo que não é do seu interesse por diversos fatores, e isso afetar tanto na saúde mental como o cuidado prestado ao outro (e.g., pessoas idosas).

**Objetivo:**

Avaliar a atitude implícita com relação a pessoas idosas entre cuidadores remunerados e não remunerados de pessoas idosas.

**Métodos:**

Conduzimos um estudo transversal com 111 cuidadores remunerados e não remunerados de pessoas idosas que residem na cidade de São Carlos-SP, Brasil e região. Os cuidadores preencheram medidas para avaliar a atitude implícita, o impacto do cuidado, sintomas depressivos e um questionário para a caracterização sociodemográfica e do cuidado. Com base no escore do teste de atitude implícita, os cuidadores foram divididos em três grupos: “atitude implícita neutra”, “atitude implícita positiva” e “atitude implícita negativa”.

**Resultados:**

Houve predominância de cuidadores do sexo feminino (91.0%). A idade dos cuidadores variou entre 19 e 76 anos (=46; [*interquartile range*-IQR=33-57] anos). Do total da amostra do presente no estudo, 63.1% dos cuidadores remunerados e não remunerados tinham atitude implícita negativa em relação à pessoa idosa e apenas 12,6% tinham atitude implícita positiva.

**Conclusão:**

Os cuidadores de pessoas idosas apresentam atitude implícita negativa com relação a pessoas idosas. Estudos futuros que avaliem o impacto da atitude implícita na relação cuidador-receptor do cuidado são necessários.

## INTRODUCTION

Providing care for older people has become commonplace throughout the world due to the increase in the population of individuals 60 years of age or older. This increase is accompanied by concerns related to health issues as well as age-related prejudice, known as ageism^1^. Ageism may be exhibited in the form of explicit and implicit attitudes^2^. Unlike explicit attitudes, implicit attitudes are often spontaneous and/or automatic, without the aid of the conscious mind^3^. Such responses are based on individual beliefs^4^. Explicit attitudes towards older people have been investigated in caregivers of older people^5^ as well as healthcare providers^6^. Implicit attitudes have been investigated in individuals in the community^7^ ^,^ ^8^ as well as students in the health field and healthcare providers^9^, and the results indicate negative attitudes towards older people. However, one study showed that having contact with older people can diminish negative implicit attitudes^10^. As caregivers have daily contact with their care recipients (i.e., older people), the same is believed to occur in this population. This is the first study to seek to identify the implicit attitudes towards older people among paid and unpaid caregivers of older people. The main hypothesis is that paid and unpaid caregivers of older people have positive implicit attitudes towards older people.

## METHODS

### Study design, participants and ethical aspects

This study received approval from the Human Research Ethics Committee of the Universidade Federal de São Carlos (certificate number: 2.498.312). A quantitative cross-sectional study with a convenience sample was conducted in the city of São Carlos and surrounding region in the state of São Paulo, Brazil, with the participation of 111 paid and unpaid caregivers of older people. Caregivers who met the following eligibility criteria were included: age≥18 years; and being a paid or unpaid caregiver of an older person (≥60 years of age). Caregivers who had less than three months of experience with providing care and those who provided care for less than four hours a day were excluded. These criteria were based on other studies conducted in Brazil involving caregivers of older people^11^ ^,^ ^12^. Written informed consent was obtained from all participants before answering the instruments employed for data collection.

### Instruments and measures

#### Implicit attitude test

This test is used to investigate implicit attitudes related to the preference for younger people or older people^13^. The test is composed of five blocks: three training blocks (i.e., 1^st^, 2^nd^ and 4^th^) and two test blocks (i.e., 3^rd^ and 5^th^) and is conducted on a computer, with a maximum response time of five minutes. The score ranges from -2.0 to +2.0. Scores up to 0.15 reveal no preference, 0.16 to 0.34 denotes a low preference, 0.35 to 0.65 denotes a moderate preference and ≥0.65 denote a strong preference^13^. This measure has a Cronbach’s alpha coefficient of 0.90^13^. For a better understanding of the test, see: https://implicit.harvard.edu/implicit/brazil/takeatest.html. Caregivers who had a score ranging from -0.15 to 0.15 were allocated to the “neutral implicit attitudes” group, those with a score ranging from -0.16 to -2.0 were allocated to the “positive implicit attitudes” group and those with a score ranging from 0.16 to 2.0 were allocated to the “negative implicit attitudes” group.

#### Zarit Burden Inventory

This scale is used to investigate the impact of providing care on paid and unpaid caregivers of older people and is composed of 22 items. The score ranges from 0 to 80 points. Higher scores indicate greater impact. The version validated for use in Brazil was employed, which has good reliability and internal consistency, with a Cronbach’s alpha coefficient of 0.87.

#### Patient Health Questionnaire

This questionnaire was used to investigate depressive symptoms considering the criteria of the Diagnostic and Statistical Manual of Mental Disorders (DSM-5). The total sum of items can reach up to 27 points. Higher scores indicate a greater occurrence of depressive symptoms. The Brazilian version used in the present study has a Cronbach’s alpha coefficient of 0.89.

#### Functional Assessment Questionnaire

This questionnaire is used to investigate the degree of functional dependence with regard to instrumental activities of daily living in older people. The score of the ten items is summed (range: zero to 30 points). Higher scores indicate a greater degree of dependence. The Brazilian version used in the present study has adequate internal consistency, as indicated by Cronbach’s alpha coefficient (0.95).

### Procedures

This study was conducted between March 2018 and January 2019. The participants were recruited though public calls on the social media of the authors’ research group (i.e., Research Group on Mental Health, Cognition and Aging - ProViVe). Caregivers who met the eligibility criteria signed the statement of informed consent and then completed the data collection instruments described above as well as a questionnaire designed by the researchers to characterize the sample in terms of sociodemographic variables (i.e., age, sex, schooling and marital status) and aspects related to the care provided (i.e., duration of care in years, days per week and hours per day, type of caregiver [paid or unpaid] and whether the caregiver resided with the care recipient). The data collection instruments were completed in an average of 40 minutes.

### Statistical analyses

Data analysis was performed with the aid of the Statistical Package for the Social Sciences (SPSS) program, version 25.0. The data are described in the Results section and Table 1 and expressed as median (), interquartile range (IQR) and percentage. The Kolmogorov-Smirnov test demonstrated that the data had non-normal distribution. Comparisons among groups (i.e., “neutral implicit attitudes”, “positive implicit attitudes” and “negative implicit attitudes”) were performed using the chi-squared test and Kruskal-Wallis H test. Pairwise comparisons of continuous variables that differed between groups were performed using the Mann-Whitney U test. For all analyses, a 5% significance level was adopted (p<0.05).

**Table 1.** Sociodemographic, care-related and clinical characteristics of caregivers of older people divided into three groups (i.e., neutral, positive and negative attitudes) according to score on implicit attitude test.

Variables		Total (n=111)	Neutral implicit attitudes (n=27)	Positive implicit attitudes (n=14)	Negative implicit attitudes (n=70)	χ^2^/H	p-value
*Sociodemographic*							
Age (years) ^[IQR]^		46 [33-57]	42 [30-57]	44 [34-56]	48 [32-57]	0.34	0.84
Schooling (years) ^[IQR]^		12 [12-13]	12 [12-13]	12 [4-12]	12 [11-13]	1.49	0.48
Sex ^n (%)^	Female	101 (91.0)	24 (88.9)	12 (85.7)	65 (92.9)	0.92	0.63
	Male	10 (9.0)	3 (11.1)	2 (14.3)	5 (7.1)		
Marital status ^n (%)^	Married	41 (36.9)	15 (55.6)	4 (28.6)	22 (31.4)	5.35	0.07
	Not married	70 (63.1)	12 (44.4)	10 (71.4)	48 (68.6)		
Skin color ^n (%)^	White	73 (65.8)	19 (70.4)	10 (71.4)	44 (62.9)	0.72	0.70
	Non-white	38 (34.2)	8 (29.6)	4 (28.6)	26 (37.1)		
*Aspects related to care*							
Type of caregiver ^n (%)^	Unpaid	60 (54.1)	14 (51.9)	8 (57.1)	38 (54.3)	0.11	0.95
	Paid	51 (45.9)	13 (48.1)	6 (42.9)	32 (45.7)		
Resides with care recipient ^n (%)^	Yes	41 (36.9)	10 (37.0)	6 (42.9)	25 (35.7)	0.26	0.89
	No	70 (63.1)	17 (63.0)	8 (57.1)	45 (64.3)		
Duration of care ^[IQR]^	Years	3 [1-5]	3 [1-7]	4 [1-5]	3 [0.8-5]	1.61	0.45
	Days per week	6 [4-7]	7 [4-7]	6 [4.75-7]	6 [4-7]	0.02	0.99
	Hours per day	12 [9-16]	12 [8-16]	14 [12-16]	12 [8-16]	0.70	0.71
*Clinical*							
Impact of providing care^a,^ ^[IQR]^		21 [14-30]	21 [15-29]	20 [13-35]	21 [13-31]	0.09	0.96
Depressive symptoms^b,^ ^[IQR]^		6 [2-11]	6 [2-12]	3 [1-13]	6 [2-11]	0.45	0.80
Functional capacity^c,^ ^[IQR]^		29 [19-33]	29 [19-33]	31 [23-33]	28 [19-33]	0.56	0.76
Implicit attitudes^d,^ ^[IQR]^		0.25 [0.01-0.52]	0.01 [-0.04-0.09]†‡	-0.40 [-0.68-0.28]†§	0.43 [0.28-0.61]‡ §	80.62	0.01*

Notes: a: Zarit Burden Inventory (higher scores indicate greater impact of providing care); b: Patient Health Questionnaire (higher scores indicate more depressive symptoms); c: Functional Assessment Questionnaire (higher scores indicate greater dependence); d: Implicit Attitude Test (higher scores indicate greater implicit attitudes); : median; *p≤0.05; IQR: Interquartile range; †‡§: Comparison between groups, Mann-Whitney - U test.

## RESULTS

Table 1 displays the data for the characterization of the participants. Women predominated in the sample (91.0%). Median age was 46 years (range: 19 to 76 years), with an interquartile range of 33 to 57 years. The difference in implicit attitudes among the groups was statistically significant (p=0.01). The caregivers in the “negative implicit attitudes” group had a higher score compared to the other groups. A total of 63.1% of the paid and unpaid caregivers had negative implicit attitudes towards older people and only 12.6% had positive implicit attitudes.

## DISCUSSION

This study investigated implicit attitudes on the part of paid and unpaid caregivers of older people. The results of the study did not confirm the hypothesis. This finding is similar to data reported in previous studies conducted with other populations^7^ ^,^ ^8^. The group of caregivers with negative implicit attitudes towards older people had a score of 0.43, indicating a moderate negative implicit attitude^13^. This finding is in agreement with data from previous studies conducted with individuals of the community^8^ and healthcare providers^14^. Studies have demonstrated that men (compared to women)^8^ and middle-aged individuals (i.e., 40-67 years)^7^ have negative implicit attitudes towards older people. In the present study, most caregivers in the group with negative attitudes were women with a median age of 48 years. Although no differences in these variables were found among the groups, other factors, such as the serious health conditions^15^, feelings of abandonment^16^, communication difficulties^17^ and greater functional dependence of older people^16^ have contributed to negative implicit attitudes among healthcare providers. We hypothesize that the same may occur with caregivers of older people. These factors have significant implications for the care provided. Such issues are not easily modified. However, interventions to diminish prejudice towards older people have been conducted^18^. One study demonstrated that interactions between grandchildren and grandparents can diminish the occurrence of negative implicit attitudes^10^, which does not seem to be the case with caregivers. However, one should bear in mind that contact between caregivers and care recipients goes beyond a casual interaction, as caregivers perform the function of providing health care (e.g., giving baths, changing clothes, etc.). Further studies are needed to understand the factors that lead caregivers to develop implicit attitudes towards older people so that effective interventions can be established to minimize these negative implicit attitudes. A recent study with a Japanese population of individuals 16 years of age or older demonstrated that an intervention involving virtual reality with a focus on implicit attitudes towards dementia improved these attitudes on the part of the younger participants, but not on the part of the older people^19^. However, more interventions are needed, as providing care for an older person may be necessary for other reasons (e.g., functional dependence). Moreover, many older people in Brazil provide care for other older people^20^. The Pan American Health Organization describes interventions (e.g., educational and/or intergenerational contact) to minimize the occurrence of ageism^21^. Interventions with a focus on empathy, which have been used with caregivers^22^ as well as paid and unpaid caregivers of older people in Brazil^23^, may constitute a strategy to minimize this attitude not only among caregivers, but also among other healthcare providers who work with older people.

In conclusion, the findings of the present study demonstrate that caregivers of older people have negative implicit attitudes towards older people. Studies with a larger sample size and longitudinal design are needed to enable a more comprehensive understanding of the implications of these negative implicit attitudes for the care provided to older people. It is also important to understand the impact of these attitudes on the mental health of caregivers. Considering the significant increase in the older population throughout the world, interventions and public policies are needed to minimize the occurrence of ageism. Lastly, although this is the first study to assess the implicit attitudes of paid and unpaid caregivers of older people, some limitations need to be considered, such as the cross-sectional design, which does not enable the establishment of causality, and the specific sample of caregivers from one region of Brazil, which has implications for the generalization of the findings. However, this study can assist in the guidance of future studies involving caregivers of older people. Moreover, the findings underscore the importance of the use of performance measures (e.g., Implicit Attitude Test) to assess this construct, as many caregivers may answer self-report measures based on what is socially expected.

## DATA AVAILABILITY STATEMENT

Due to the nature of this research, the participants did not agree for their data to be shared publicly. Therefore, no supporting data is available.
